# Oral health assessment in a sample of displaced people as a result of the war in Syria in Damascus city: results of non-profit initiative

**DOI:** 10.1186/s12903-021-01874-9

**Published:** 2021-10-15

**Authors:** Rudwan Kazwini, Tarek Kasem, Noor Ewaz Ali Alhuda, Marwah Albarshah, Dania Subeh, M. H. D. Bahaa Aldin Alhaffar

**Affiliations:** 1 Department of Periodontology, Faculty of Dental Medicine, Damascus University, Damascus, Syria; 2 Department of Pediatric Dentistry, Faculty of Dental Medicine, Hama University, Hama, Syria; 3 Department of Fixed Prosthodontics, Faculty of Dental Medicine, Damascus University, Damascus, Syria

**Keywords:** Displaced people, Oral health, War on Syria, DMFT index

## Abstract

**Background:**

War on Syria extended for a long time and resulted in significant impacts on various aspects, one of these aspects was displaced people crisis, and thus its impact on complete neglecting of oral health despite of its importance and impacts on the general health. This study aims to assess the oral health of the displaced Syria sample as a result of the war on Syria.

**Methods:**

The sample included 118 patients of displaced families from different regions and cities to shelters in Damascus city, and the study included 118 control samples from the Faculty of Dentistry, Damascus University. The non-profit initiative team included 20 dentists of all dental specialties. Oral health was assessed using DMFT index. Data were analyzed using SPSS V.22 in comparison with the gender of the patients, age groups, and socioeconomic status. Finally, the number of the treatment provided by the non-profit initiative was collected and presented in the study.

**Results:**

No significant differences found in the DMFT index between children and adults (*P* = 0.750), DMFT value ranged between (0 and 11) with a mean value (2.4). The total DMFT value for adults was (2.77), while for children (2.12). Also, no statistical difference was found between males and females (*P* = 0.688). While the control group had an average DMFT value of (2.37), the difference between the displaced and control samples was not significant. Over 200 dental treatments were provided by the team.

**Conclusion:**

This study concluded that the DMFT value is high among the displaced people as one of the consequences of the war on Syria, however, no significant difference was found when the results of the displaced sample was compared to a control sample.

## Background

The war on Syria that began in 2011 had a great impact on various aspects inside Syria [1]. As the Syrian territories that were not subject to the Syrian state had become one of the most dangerous places for healthcare providers, and recent studies have found that hundreds of health care workers have been killed and/or tortured, and many health facilities have been destroyed [2]. Fouad et al. 2017 found that during 7 long years of war on Syria [3, 4], more than 6 million people have migrated inside Syria, in the largest displacement crisis in the world happened, this is equivalent to six out of ten Syrians who have been displaced from their homes [5]. The displaced Syrians faced difficulties and obstacles in obtaining necessary health care and medicine in times of displacement [6]. Although the actual war on Syria ended nearly two years ago, and despite all the continuous measures taken by humanitarian organizations and governmental organizations to alleviate these difficulties and obstacles, there is still a group of displaced people present in the shelters until now due to their difficult economic conditions [7].

The war on Syria led as well that Oral health has also been completely ignored by the displaced, despite its importance and impacts on public health. This happened due to the complexity and relatively high cost of dental treatments, which made it very difficult for the displaced to obtain them. According to research published in 2019 AD, the rate of tooth decay during the war in Syria was higher than expected, and the prevalence rate among children was 79.1%, with an average of DMFT (2.03) [8]. another study assessed oral health among children in comparison with socio-economic status found that the average DMFT among children was (3.36) with 86% had at least one dental problem [9]. Another study documented the oral health status among children in refugees' camps in Jordan and found that Syrian refugee children in Jordan have high levels of unmet dental needs [10].

However, oral health is a very important indicator to a person’s general health status [11]. It also positively adds to a person's mental and social physical health. Knowledge of the current state of oral health in one community has a great role in addition to its impact on any treatment or preventive interventions that can be widely adopted, whether in educational curricula and teaching strategies or even in oral health awareness programs [12, 13].

Dental caries consider one of the most common disease worldwide, and considers a public health problem in many countries [14]. Studies showed that poor oral health also leads to various systemic diseases such as cardiovascular and respiratory diseases [15, 16], in addition of being the number one cause of tooth loss worldwide. The prevalence of dental caries varies between countries, as it is more prevalent in less developed countries due to poor socio-economic status [17], as well as environmental factors [18]. Therefore, it is important to study oral health on a sample of the displaced as a result of the war on Syria in Damascus.

Asnan-Lulu Initiative is a volunteer project within the Peace Organization that aims to examine oral health, provide dental treatments and raise awareness for the displaced people as a result of the war on Syria inside Damascus city by 20 dentists specialized in all dental specialties.

### The aim of the research

Evaluating the oral health of the displaced people (adults and children) as a result of the war on Syria within the city of Damascus through studying of the DMFT index and mentioning the number of treatments that have been provided (through the Asnan-Lulu Initiative).

## Methods

### Study design

A survey study to assess oral health, carry out treatment procedures, and raise awareness of oral health and tooth brushing for internally displaced people due to the war on Syria within the city of Damascus in two dental centers from October 2019 until November 2019.

### Sample size

The sample included 118 patients of displaced families from different regions and cities to shelters in Damascus and Damascus suburbs. Most of the patients in the sample were displaced from Yarmouk Camp and Deir Ezzor province.

The control sample also included 118 patients from the Faculty of Dentistry, Damascus university. Those patients share the same socioeconomic status with the displaced sample, however, only patients who has not been displaced were included in the control sample. Patients at the faculty of dentistry are randomly distributed which ensure the randomization of the data in control group.

### Inclusion criteria

1. Internally displaced families to shelters holding identification numbers in Damascus and Damascus suburbs.
2. Internally displaced families to shelters who cannot afford dental treatments’ costs.

### Exclusion criteria

1. Internally displaced families from their home to another home in another location.
2. Internally displaced families who have the financial ability to afford the dental treatments costs.

The team consisted of 20 doctors from all dental specialties (9 periodontics, 7 pedodontics, 2 endodontics, 1 prosthodontic, 1 oral medicine), where the oral medicine specialist diagnosed all patients and recorded each patient's data on his own diagnostic card (An adult or a child) then the specialist performed the required dental treatment and then made awareness about oral health. Oral health was assessed based on Decayed, Missed, and Filled Tooth (DMFT) index. All data were analyzed by a statistician.

### Control group

The reason behind having control group is to compare the results of the displaced sample with non-displaced sample (control group) which will give better insight about the effect of displacement during the period of the Syria war on oral health in particular.

The control group was collected from the dental clinic at Damascus university for many reasons, the patients at the faculty of dentistry are required to fill their information on a data collection paper, which made the data collection for control group easier. The second reason is related to the ethical approvals, as the ethical approval obtained from the universities and only allowed to collect data from the patients in the faculty, therefore can't collect data from private clinic. The third reason is that the patients at the faculty of dentistry share the same socioeconomic status as the displaced sample, they can't afford private clinics, they seek for free and quality treatments. Finally, most of the research team works at the university as higher education students (MSc, PhD students) which will guarantee the quality of data collected, and the quality of treatments provided.

### Examination tools

- Dental mirror.
- Explorer.
- Tweezers.
- Personal protecting equipment (PPE).

### Statistical analysis

Data were collected and added to Excel database. SPSS v.22 was used to analyse the data, both descriptive and inferential statistics was used. Shapiro–Wilk test was used to ensure the normality of the data, *T*-test, were used to study the significant difference between the research variables.

## Results

The research sample consisted of 118 male and female patients from the displaced sample, age ranged from (4 to 60 years), and the average age was (22.7 years). The number of males in the sample was 54 (45.7%), and the number of females 64 (54.3%). The control sample also consisted of 118 patients who were identical in terms of demographic variables but were not internally displaced.

The sample was divided into two groups according to ages, the group of children (< 14 years old), and the group of adults (older than 14 years) according to the recommendations of the World Health Organization (WHO) in the Directory of Medical Research in the field of dentistry, where the number of children reached 52 (44.1%), and the number adults 66 (55.9%).

Oral health instructions were given to each patient (100%), they were instructed to the correct brushing method, the number of times and duration of brushing, in addition to oral habits that harm the teeth and how to avoid them.

Oral health was assessed using the DMFT index in the children and adults. The number of broken, repaired, and missing teeth was calculated, and the total value of the DMFT index was calculated. Where the value of the decayed teeth ranged between (0 and 5) teeth and the mean value were (0.9), the number of missing teeth ranged between (0 and 6) and the mean value was (0.8), while the number of restored teeth was between (0 and 5) with mean value. (0.7). The DMFT index value was between (0 and 11) with a mean value of (2.4) (Table 1).

**Table 1:** Oral health assessment using DMFT index for the displaced sample in comparison with age and gender (sample size = 118, year of data collection 2019)

Descriptive analysis for the whole sample						Analysis according to age			Average DMFT according to gender^a^
	N	Min	Max	Mean	SD	Group	Mean	*P* value^a^	
DMFT index	118	.00	11.00	2.48	2.377	Children	2.12	0.225**	Males = 2.28 Females = 2.65 *P* = 0.688**
						Adults	2.77		
Extracted teeth	118	.00	5.00	.94	1.347	Children	1.08	0.000*	
						Adults	.83		
Missing teeth	118	.00	6.00	.83	1.339	Children	.42	0.657**	
						Adults	1.17		
Filled teeth	118	.00	5.00	.70	1.096	Children	.62	0.750**	
						Adults	.77		

^*^Significant difference

^**^No significant difference

^a^Independent samples *T*-test

The level of oral health was studied according to age groups (children, adults). The value of carious teeth D was greater in the children's group (1.08), while it was in the adult group (0.83).

As for the missing teeth M, restored teeth F, and the total value of DMFT between the two age groups in the adult group, the total value of DMFT was greater in adults (2.77), while in children (2.12).

Only a significant difference was observed between the number of missing teeth between the two age groups (*P* = 0.000), while no significant difference was observed between the rest of the variables (Table 1).

Independent samples *T*-test was used to study the relationship between the value of the DMFT and the sex variable, and the test result showed that there is no significant difference between males and females in terms of the value of DMFT, as (*P* = 0.688). However, DMFT was higher for females than for males (Table 1).

Numerous treatments were provided through the initiative, and the number of free treatments provided during the life of the initiative reached (203) treatments, as patients in total received a number of treatments ranging between (1 and 6), and the mean value was (1.7), as each patient received At least two treatments, in addition to awareness and topical application of fluoride, if necessary. The largest number of treatments provided was restorative treatments (59) treatments, followed by treatments with tooth extraction (54), where the team tried to preserve the teeth by restoring them, but as noted from the table, a large number of treatments were extractions, because the teeth were not restorable due to bad Oral hygiene. The total number of Scaling and root planning procedures, endodontic treatments, and pulpotomy was (44, 32 and 14) respectively.

The control group consisted of 118 people to compare the DMFT with a sample of displaced families, the data for comparing sample were collected from external clinics of the faculty of dentistry, where large number of patients come from different areas of Damascus. Table 2 shows the number of each value of DMFT index in the control sample, as the mean value was (2.37), and ranged between (0 and 10). Mean value of extracted teeth was (1.8), filled teeth (3.43), missing teeth (1.75).

**Table 2:** Oral health assessment using DMFT index for control sample (sample size = 118, year of data collection 2019)

	N	Minimum	Maximum	Mean	SD
Number of extracted teeth	118	0	4	1.8	1.23
Number of missing teeth	118	0	3	1.75	1.43
Number of filled teeth	118	0	19	3.43	0.98
DMFT value	118	0	22	2.37	1.4

Comparison between displaced and control sample in the DMFT value				
	Mean DMFT	SD	*P*-value*	significance
Displaced sample	2.48	2.3	0.087	Not significant
Control sample	2.37	1.4		

^*^Independent samples *T*-test

Table 2 shows the result of independent samples *T*-test to study the existence of a statistical difference between each of the displaced sample and the control sample in terms of DMFT index, the test showed there was no statistical difference between each of the displaced sample and the non-displaced sample as (*P* = 0.087), but the displaced sample showed a higher DMFT index value leading to a lower level of general oral health comparing to non-displaced sample.

## Discussion

There is no doubt that the Syrian war has directly affected the health care system in Syria [19], over 50% of the health care facilities are out of service, and over 70% of the healthcare providers has left the countries seeking safety in other places [1].

Oral health is considered as a secondary objective when providing emergency healthcare services for any displaced or refugee population [9], despite the fact that oral health can reflect the overall health condition of any patients, and can affect the quality of life for adults and children as pain from oral sources have high prevalence among displaced populations [9]. Therefore, it was necessary to carry out this study to find out the impact of the war in Syria on the oral health of displaced adults, in addition to comparing the information that we had with the existing information on the DMFT index in children.

This research evaluated the oral health using DMFT index in displaced sample in Damascus city–Syria, the results of this study found that the overall oral health among the displaced sample is bad, however, when compared to a control group similar results are found, which can show that the low oral health status is not directly related to the effect of displacement, and it is related with the low economic status and the fact that people in Syria can't access dental health services as it is considered very expensive for the majority of the Syrian population.

In this study, the average value of the total DMFT indicator in adults was (2.77), while in children (younger than 14 years) (2.12), which is higher than the rate recommended by WHO [20]. There was no significant difference in the value of DMFT between the two genders, except that the value of DMFT was higher in females compared to males.

Previous study evaluated the DMFT values for two decades in Syria from 1980 to 1999 by Beiruti and Tayfour showed that children aged 5 years had dmft value of (4.7–5.2), and for children aged 12 years (1.9–2.3); Where these values of DMFT are lower compared with the values of DMFT in this study, which recorded a total value in children (2.12) [21].

In another article to study the effect of socioeconomic status on DMFT values in Syria in 2019, AlHaffar found that the results were in children of 12 years old (3.36); These DMFT values are higher compared to DMFT values in this study which recorded a total value in children (< 14 years) (2.12), however, the sample size in Al-haffar study was bigger which can indicate the reasons for the difference in the values [22].

An oral health study among children in Damascus during the war in Syria in 2019 showed that DMFT results are (2.03 ± 1.81) [23], while another study in latakia for the same age group found that DMFT recorded an average of (2.35) [24].

While comparing the results of this study with the studies conducted in the Arab world, we find:

A study in Dubai in 2013 by Al-Mashhadani and Khoury showed the following values: Children aged 6–5 years The DMFT was (3. 87), children aged 12–15 years old, the DMFT was (1.83; while the value of DMFT in this study was in children (< 14 years) (2.12) [25].

On the other hand, several studies studied the oral health among a sample of refugees, in Jordan, children have high levels of dental caries and oral problems and they are not receiving enough dental treatments [10], while another study found that the decreased financial support for refugees from both governmental and non-governmental sources led to increase in the number of dental problems among refuges, and inhibited the activities of dental initiates which considered the only source of dental treatments for the population living in refugees camps [26], while another research in refugees camps in Spain found that the prevalence of dental caries among refugees is over 75% and the DMFT index was over 3.2 which point out the need to provide dental treatments among refugees and population affected from the war and displacement [27].

Moreover, during the process of this study, a team of dentist provided dental treatments for the displaced population sample, most of the treatments were provided in the initiative were restorative treatments and extractions, the number of restorative treatments reached (59) treatments, followed by extractions (54) treatments, as an attempt was made to preserve the teeth by restoring them, while the teeth that needed extraction it was due to the long period of poor oral hygiene that the patients suffered of, and this indicates the depth of suffering experienced by individuals and the resulting oral health neglecting.

Finally, the study focused on evaluating the oral health status among internal displaced population inside Syria, as no previous study focused on this group, the results of this study can be used for documentation of the effect of the current crisis of oral health, and to compare the current results with future research on oral health.

## Conclusion

Within the limits of this study we can conclude that the oral health status of the internally displaced sample is poor, however, when compared with a control group of non-displaced sample, similar results were found regarding oral health, which indicate that the main reason of bad oral health is the low socioeconomic status, and the displacement can rapidly change the socioeconomic status of the population, therefore, more attention and dental care should be provided to the displaced population inside Syria.

### Recommendations and suggestions

The results of this study addressed the importance of oral health especially among people who had rapid change in their socioeconomic status, therefore, the following recommendations are important to improve the oral health:

1. Regular oral examination and emergency dental treatments for the displaced population.
2. cooperate with schools and institutions that deal with children to provide the best oral and dental health awareness services
3. provide preventive treatments and applying fluorinated materials to prevent decay among children of displaced sample.

## Data Availability

The datasets used and/or analysed during the current study are available from the corresponding author on reasonable request.
